# Effect of protein/essential amino acids and resistance training on skeletal muscle hypertrophy: A case for whey protein

**DOI:** 10.1186/1743-7075-7-51

**Published:** 2010-06-17

**Authors:** Juha J Hulmi, Christopher M Lockwood, Jeffrey R Stout

**Affiliations:** 1 Department of Biology of Physical Activity, University of Jyväskylä, P.O. Box 35, 40014 Jyväskylä, Finland; 2 Department of Health & Exercise Science, University of Oklahoma, Norman, OK 73019, USA

## Abstract

Regardless of age or gender, resistance training or provision of adequate amounts of dietary protein (PRO) or essential amino acids (EAA) can increase muscle protein synthesis (MPS) in healthy adults. Combined PRO or EAA ingestion proximal to resistance training, however, can augment the post-exercise MPS response and has been shown to elicit a greater anabolic effect than exercise plus carbohydrate. Unfortunately, chronic/adaptive response data comparing the effects of different protein sources is limited. A growing body of evidence does, however, suggest that dairy PRO, and whey in particular may: 1) stimulate the greatest rise in MPS, 2) result in greater muscle cross-sectional area when combined with chronic resistance training, and 3) at least in younger individuals, enhance exercise recovery. Therefore, this review will focus on whey protein supplementation and its effects on skeletal muscle mass when combined with heavy resistance training.

## Review

Net protein balance (NPB) is defined as muscle protein synthesis (MPS) minus muscle protein breakdown (MPB), or NPB = MPS - MPB. Thus, a significant rise in skeletal MPS (anabolism) and/or reduction in MPB (catabolism), such that NPB remains positive can result in increased skeletal muscle mass accretion. Conversely, a negative NPB, arising from a reduction in MPS and/or increase in MPB, will result in a loss of skeletal muscle. It has clearly been demonstrated that an acute bout of heavy resistance exercise - intermittent exercise of repeated short, high-intensity (60-90% 1 RM) bouts [1] - stimulates a significant increase in MPS. However, NPB remains negative due to a concomitant rise in MPB when resistance exercise and recovery occur under fasted conditions [2-4]. Pre- or post-exercisise ingestion of protein (PRO) or essential amino acid (EAA) can increase MPS and result in a positive NPB [3,5-11]. Furthermore, the majority of studies in humans suggest that PRO/EAA ingestion in the context of a resistance training session can enhance skeletal muscle hypertrophy in response to chronic resistance training [12-20]. More specifically, PRO/EAA intake around the time of resistance exercise, as opposed to nutrient ingestion at times distal to exercise, may be more beneficial for promoting muscle hypertrophy [21,22].

Milk contains two categories/fractions of PRO - whey and casein. About 20% of the total PRO in commercial bovine milk comes from whey [23-25]. Direct and indirect evidence suggests that whey may be an especially suitable PRO to be used in conjunction with resistance exercise to stimulate muscle hypertrophy [9,20,26,27]. If correct, this may contribute to the observed widespread use and sales of whey PRO amongst sports nutrition consumers. This review will, therefore, focus on whey PRO supplementation and resistance training as it pertains to muscle mass adaptations in healthy adults. Some general effects of PRO/EAA will, however, be reviewed first.

## Effects of PRO/EAA supplementation on MPS and skeletal muscle hypertrophy

Heavy resistance training has a well documented positive effect on skeletal muscle size [20,28-31], whereas ingestion of sufficient amounts of PRO/EAA also plays an important role in muscle adaptations. For example, in young men, PRO/EAA supplementation in combination with resistance training has been shown to significantly increase myofiber cross-sectional area greater than a non-energetic or carbohydrate placebo [12-15]. Additionally, PRO/EAA has been shown to be more effective than carbohydrate or non-energetic placebo at increasing lean or fat-free body mass and whole muscle cross-sectional area [14-20,32]. Contrary to the aforementioned, some studies have reported that PRO/EAA ingestion provides no significant effect on myofiber size or lean body mass during resistance training [33-36]. Overwhelmingly, however, in young males, PRO/EAA has been shown to positively augment the physiological adaptation to exercise. For instance, Andersen et al. [12] investigated the effects of a mostly whey-containing PRO blend versus an isoenergetic carbohydrate, consumed before and after resistance exercise for 14 weeks, in previously untrained young men. Only the PRO group showed type I and II myofiber hypertrophy (18% and 26%, respectively). Similarly, Hartman et al. [15] reported that consumption of fat-free milk after resistance exercise, for 12 weeks, increased lean mass and type II myofiber crossectional area more than consuming soy or carbohydrate in previously untrained young males. Candow et al. [17], on the other hand, found that both whey and soy PRO increased lean tissue mass more than an isocaloric carbohydrate placebo in healthy, young men. Hulmi et al. [20,37] has also reported that combined PRO ingestion and resistance training in previously untrained males, following an *ad libitum *diet, provides an augmented physiological adaptation to training. Specifically, ingestion of 15 g of whey PRO isolate (WPI) immediately before and after resistance exercise, for 21 weeks, increased skeletal muscle cross-sectional area [20] and seemed to accelerate increases in muscle thickness 37 more than ingestion of a non-energetic placebo. No significant effects between groups on direct measures of myofiber size were observed [37]. However, this non-effect may be due to large variation inherent within the measurement. Interestingly, the significant differences observed between groups occurred despite both groups consuming a relatively large habitual ingestion of PRO from diet alone (~1.4-1.5 g/kg bodyweight/day).

Conversely, in older males, the effects of PRO/EAA supplementation on exercise adaptation have been less clear. Verdijk et al. [36] reported no effect of casein versus a non-energetic placebo when consumed before and after resistance exercise in older men, and Godard et al. [34] showed no effect of 12 weeks of combined resistance training and low-dose EAA ingestion in older males. Similarly, Kukuljan et al. [38] reported no significant between-group differences in older males involved in 18 months of resistance training or with those also consuming fortified milk twice daily. Main effects for time were, however, noted for subjects in the milk plus exercise group for increases in lean mass and muscle cross-sectional area. Therefore, the long-term data thus far would appear to support the occurrence of a more robust MPS [39,40], or possibly altered MPB response from PRO/EAA ingestion in younger populations than older. However, this difference may not always exist, at least with large doses of PRO ingestion [41]. Katsanos et al. [39] showed that older individuals may require a higher absolute dose of leucine to effectively stimulate MPS. Additionally, PRO source and/or form may significantly affect the MPS response observed in older subjects [10]. It is evident that more long term studies comparing healthy younger and older subjects are needed, as are more studies in healthy female populations. However, the few available studies involving females seems to suggest that PRO ingestion is comparably effective within this population as in healthy young males [32].

It must also be noted that a large variability exists to resistance training only, at least in previously untrained subjects [42]. Therefore, any effect of PRO/EAA supplementation within previously untrained subjects may be overshadowed by a large standard error of means. This may be especially true when the intervention is of short duration and/or sample sizes are small. Similarly, though some body composition methods commonly used to report changes in fat-free mass - dual-energy x-ray absorptiometry or air displacement plethysmography - can accurately track changes, their respective total statistical error of measure may be too large to assess small, but significant changes between groups/interventions [43]. Such considerations should be accounted for in future trials to increase statistical power and reduce the probability of type II error confounding study conclusions.

## PRO/EAA ingestion may enhance recovery from heavy resistance exercise

Several studies support the use of EAA or PRO, when supplemented within the context of exercise, to enhance recovery of muscle function as measured ~1-4 days after heavy exercise [44-49] and to decrease proxy markers of muscle damage or soreness [44-48]. Though increased EAA availability may simply provide a more robust anabolic environment, enhanced recovery may also be explained, in part, by EAA's possible anti-catabolic effect of decreasing whole body myofibrillar protein degradation in the days following strenuous exercise [50]. However, more direct evidence is needed to support the hypothesis that inhibiting catabolism improves recovery and adaptations from heavy exercise. Interestingly, EAA concentration alone likely cannot entirely explain the improved rate of recovery for measures of peak isometric torque observed by Buckley et al. [46]. Specifically, when previously untrained subjects consumed either 25 g WPI or 25 g of its extensive hydrolysate (WPH), peak isometric torque was fully recovered by 6 hours post-fatiguing eccentric exercise for subjects consuming the WPH, whereas recovery was still significantly depressed by 24 hours post-exercise in both the WPI and placebo groups [46]. However, no differences between groups were observed for muscle soreness or proxy measures of muscle damage (e.g., creatine kinase) for up to 24 hours post-exercise. Thus, more research needs to be conducted to assess changes over a greater time period.

In the case of whey PRO or its hydrolysates, whether consumed in the absence or presence of carbohydrate, one explanation may be that whey provides a post-exercise insulin response [51,52] (or augments the response from carbohydrate) such that NPB can be slightly smaller and glycogen resynthesis occurs more rapidly; thus, the recovery from exercise may be enhanced. Power et al. [52] showed that 45 g of WPI or its extensively hydrolyzed WPH yielded similar rates of gastric emptying when consumed by healthy adults under fasted conditions. However, 3-hour area under the curve and peak insulin response for the WPH was found to be 43% and 28% greater than for WPI, respectively. Similarly, Morifuji et al. [53] reported that rat skeletal muscle glycogen concentrations were significantly greater two hours after completing a glycogen-depleting exercise bout when carbohydrate+WPH was consumed immediately post-exercise (CHO+WPH > CHO+WPI ≈ CHO+BCAA > CHO+CPH ≈ CHO; CHO, BCAA and CPH refer to carbohydrate, branched-chain amino acids, and casein PRO hydrolysate, respectively). Interestingly, Morifuji et al. [54] identified several BCAA-containing dipeptides from hydrolysis of the whey fraction, beta-lactoglobulin, that were shown to significantly increase glucose uptake in L6 myocytes and glycogen concentrations in isolated skeletal muscles. Whether these results can be duplicated in humans and can be achieved in response to heavy resistance training remains to be seen.

## Mechanisms underlying the effects of PRO/EAA ingestion on MPS and recovery

High-quality PRO such as whey effectively stimulates the synthesis of myofibrillar and sarcoplasmic protein fractions in muscle under resting conditions and in response to resistance exercise [9]. Possibly the most important component of high-quality PRO may be its large concentration of BCAAs. Specifically, BCAAs [55,56] appear to elicit similar mTOR signaling as occurs in response to intact whey [37] and casein hydrolysate [57] when provided in the context of a bout of resistance exercise. However, based upon animal and cell culture studies, only leucine is required to induce protein synthesis [58-60] and mTOR signaling [61]. *In vivo *human studies are needed to validate this hypothesis. The precise mechanism by which amino acids activate mTOR may involve increased intracellular Ca^2+^, which in turn activates a class III PI3 kinase human vacuolar protein sorting 34 (hVps34) [62]. In the activation of mTOR by amino acids, Rag GTPases [63,64], Rheb [65] and MAP kinase 4 [66] may also be utilized.

In theory, enhanced exercise recovery due to PRO/EAA ingestion may also be partially explained by the results of a recent study in older adults. Just one week of a low protein diet (0.5 g/kg b.w./d) *decreased *several transcript levels in muscle that relate positively to cell proliferation, and *increased *transcript levels that negatively regulate cell proliferation [67]. Moreover, gene expression of cyclin-dependent kinase 2 (cdk2), a factor that positively affects cell proliferation [68,69] and animal size [69], has been shown to increase in humans after acute and chronic bouts of resistance exercise, but only when whey PRO is ingested around training times [20,70]. Regardless of the cellular mechanisms positively affected by exercise and PRO/EAA ingestion, enhanced recovery enables greater training volume [71]. Increased training volume alone may support increased muscle hypertrophy. As more studies assessing the effects of PRO/EAA at the cellular level are conducted, a viable theory will likely surface to more directly address these issues.

## Effect of PRO/EAA timing on MPS and skeletal muscle hypertrophy

PRO/EAA intake immediately following or immediately before and after resistance exercise, as opposed to nutrient ingestion at times distal to exercise, has been reported in some studies to be more beneficial for promoting muscle hypertrophy in both previously untrained [21] and trained subjects [22]. As has been speculated (see review: [72]), one reason for the timing effect may be increased skeletal muscle circulation, and thus nutrient transport during and for a short period following exercise. Thus, there seems to exist an enhanced time-window for the uptake of amino acids and glucose into muscle [2,73-75]. If true, this appears to occur concomitantly within the time period when MPS has the greatest elevation in response to exercise [76] and may explain why early nutrient timing after exercise seems to be especially beneficial in increasing MPS (see Burd et al. [77]).

It may be possible, however, that timing of nutrient ingestion does not always significantly affect resistance training adaptations. This may be the case at least for some PRO sources or for PRO in the absence of carbohydrates. As evidence, a recent 10-week study by Hoffman et al. [78], in resistance-trained athletes, reported that PRO-alone, consumed either immediately before and after exercise or in the morning and evening, did not significantly affect strength, power, or changes in body composition. The PRO source was a blend of collagen hydrolysate, WPI and casein. Unfortunately, total daily PRO intake for the morning-evening group increased significantly more than for the pre-/post-exercise group. Therefore, it is possible that the effects observed may not be predictive for all PRO, and in fact a positive bias for the morning-evening group may have confounded the results altogether. However, it may be possible that the subjects' high degree of prior strength training experience may have also created a "ceiling effect" requisite of a greater sample size and smaller effect size to observe small, but statistically significant between-group changes. In a study in previously untrained subjects involved in eight weeks of resistance training, 70 g/d of PRO (82% as the slow digesting PRO, casein [79]) reportedly increased fat-free mass when consumed in the morning and evening (five hours after resistance exercise) as opposed to being consumed in the morning and afternoon (immediately before training) [80]. Interestingly, these results may suggest that ingestion of slow digesting PRO, such as casein, prior to a period of prolonged fasting (e.g., bedtime) could be advantageous to increasing muscle hypertrophy. However, it is also possible that the aforementioned Burk et al. study [80] more practically provides evidence to support the preferential need for post-exercise PRO/EAA supplementation, so long as previously untrained subjects consume PRO/EAA within five hours after exercise. Specifically, peak MPS response has been shown to be delayed in previously untrained individuals [81].

The majority of available data has concluded that whey or total milk PRO (whey+casein) ingestion immediately after resistance training better augments the MPS response beginning at ~1-3 hours post-exercise, compared to recovery under fasting conditions or consumption of carbohydrates or soy immediately post-exercise [5-11]. Moreover, in a fasted state, a decrease in MPS *during or immediately after *resistance exercise may be prevented by provision of EAAs before and/or during exercise [82-84]. A recent study reported no increase in *post-exercise *MPS following EAA ingestion 1 h pre-exercise as compared to exercise in the absence of EAA's [80]. In another study, positive but similar leg net amino acid balance (an indirect marker of protein synthesis) was observed post-exercise when 20 g of whey protein was ingested immediately before exercise or 1 h postexercise [5]. Contrastingly, an earlier study from the same group reported that the ingestion of EAAs and CHO immediately before resistance exercise produced a larger increase in leg net amino acid balance compared to EAA+CHO provided immediately postexercise [73]. Explanations for the apparently confounding results of the prior study [73] may be that the length of the fasting period prior to the intervention was of too long a duration and/or an artifact, such as pooling of amino acids within muscle may have occurred. Moreover, the observation of a 20-fold increase in blood flow would seem to indicate that it is unlikely the results completely represented MPS [73]. Therefore, the question remains whether post-exercise PRO/EAA ingestion alone is enough, especially under normal dietary situations (eg, non-fasted), to elicit optimal muscle anabolism in response to exercise. This probably depends at least on the timing of the last meal before exercise and possibly on the duration/intensity/type of exercise. Lastly, non-significant trends observed by Beelen et al. [83] indicate that PRO ingestion prior to exercise may slow the breakdown of whole-body protein during resistance exercise. However, even if whole-body protein breakdown is reduced during exercise, what effect if any this may have on MPB and eventually on muscle adaptation, remains to be explored. Mindful, of course, that a change in MPS contributes more to the NPB of muscle than a change in PRO degradation (NPB = MPS - MPB) after a bout of resistance exercise with or without nutrient ingestion [85].

## Effect of PRO/EAA dose on MPS and skeletal muscle hypertrophy

As few as 10 g of whey PRO has been shown to effectively stimulate MPS following resistance exercise [8]. However, the optimal PRO/EAA dose to elicit both the most dramatic rise in acute MPS and chronic muscle hypertrophy adaptations has yet to be fully elucidated. Acute studies utilizing egg PRO [86] or EAA [40] seem to indicate a plateau in MPS from the ingestion of between 20-40 g of intact PRO, or approximately 9-10 g of EAA, respectively. In agreement (to the existence of a plateau effect), no difference in mixed MPS was observed in a recent study involving the ingestion of 30 or 90 g of PRO from intact beef [87]. Whether or not such a plateau effect is predictive and independent of PRO source has yet to be determined. Similarly, subject-specific dosing and resistance training variables (e.g., volume, intensity, etc.) may also have an effect on the optimal PRO dose. Frequency of PRO dosing is also in its infancy and requires more analyses. For example, it would appear from the available acute MPS data that small (delivering ~10 g EAA), frequent (every 2-5 hrs) PRO feedings are ideal for maintaining a positive NPB in skeletal muscle, at least when consuming a fast-acting EAA source [40]. However, some studies suggest that larger, less frequent PRO/EAA containing feedings provide similar or possibly better responses on lean body mass and protein balance [88,89]. More studies are clearly needed to fully address this issue and to determine if, for example, the optimal frequency and/or dose is a protein source dependent phenomenon.

## Effect of protein source on MPS and skeletal muscle hypertrophy

The literature is relatively sparse in comparing the acute or chronic effects of PRO source on MPS or muscle hypertrophy, respectively. Greater peak leucine net balance over time (indirect estimate of net muscle protein synthesis) was observed for whey versus casein PRO when ingested 1 h post-exercise, though no significant differences in net phenylalanine balance were observed [90]. A recent study by Tang et al. [11] showed that whey PRO appears to promote a larger MPS response than either casein or soy, at least during the first three hours post-ingestion; both at rest and after resistance exercise in young, healthy males. In fact, at rest, whey was shown to be ~93% and ~18% more effective than casein and soy, and whey was ~122% and ~31% more effective than casein and soy following exercise, respectively. It should be noted that WPH was used in the aforementioned study, whereas the soy and casein were intact. It may be probable, though, due to the large differences observed between the proteins, that WPI or whey PRO concentrate (WPC) would have provided similar results. However, recent results [46,53] would seem to indicate that at least some differences in acute effects may occur in response to WPH versus intact whey. Whether such acute differences between the intact and hydrolysed PRO sources summate over time and reveal significant chronic effects is an area of needed research.

Few long-term studies comparing different PRO sources have been published thus far. Hartman et al. [15], for example, observed greater gains in fat-free mass and muscle hypertrophy in response to 12 weeks of resistance training with ingestion of fat-free milk versus soy. Additionally, Cribb et al. [26] reported a five-fold increase in lean body mass from supplementation with hydrolyzed whey PRO isolate versus intact casein, when combined with 10 weeks of resistance training in recreational bodybuilders. Candow et al. [17] and Brown et al. [91], however, reported no significant differences on changes in body composition in response to combined resistance training and either whey or soy PRO ingestion. Kalman et al. [92] also found no differences between groups for lean body mass when examining the effects of 12 weeks of resistance training combined with 50 g/d PRO supplementation from either a whey PRO blend (50:50 ratio of WPI:WPC), soy concentrate, soy isolate, or a 50:50 ratio of soy isolate and the whey blend. Although interesting, some limitations within the aforementioned pilot study [92] do exist, as is evident by the author's own statement that "the data is considered inferential and not conclusive". That is, the sample size was too small to yield high statistical power. Indeed, of many of the studies to date, statistical power has likely not been high enough to adequately differentiate the effects between PRO source. A meta-analysis of studies (>200 subjects) in which PRO supplementation was used to promote muscle hypertrophy in combination with resistance training, was recently published that addresses this issue of statistical power [93]. Specifically, Phillips et al. [93] discovered that whey PRO elicited significantly greater gains in lean body mass than soy or carbohydrate, but was not significantly different than the effects of milk PRO.

One explanation for the superiority of dairy PRO at increasing skeletal muscle mass may be explained by the results observed by Fouillet et al. [94] that reported significantly greater peripheral nitrogen retention from milk PRO than soy PRO; soy was found to have a more profound effect on splanchnic protein synthesis. Additionally, the rate at which amino acid concentrations rise may significantly affect MPS [11,95]. For example, whey is absorbed rapidly and is relatively short-acting, quickly and vastly increasing whole body protein synthesis (estimated by non-oxidative leucine disposal) compared to casein [79,96]. Paradoxically, it has been proposed that whey may have a negligible, or at least smaller decreasing effect on whole body protein breakdown (estimated by endogenous leucine rate of appearance) than may occur in response to slower acting PROs such as casein [79], but not all studies agree with this [97].

The effects of PRO sources other than milk or soy, on muscle hypertrophy, are both fewer in number and more equivocal. For example, Campbell et al. [98] showed that a meat-containing, as opposed to a lacto-ovovegetarian diet provided greater gains in fat-free mass and skeletal muscle mass during resistance training in older men. These results were refuted by a later study [99]. Though lean beef and other high quality, PRO-rich foods (e.g., eggs) can induce a robust increase in muscle protein synthesis [86,87,100], clearly more studies comparing different PRO sources are needed to assess chronic adaptations when combined with resistance training.

## Whey protein

Human milk contains two categories/fractions of PRO - whey and casein - in an ~90:10 ratio during early lactation; whereas the ratio shifts to approximately 60:40 and 50:50 during mature and late lactation, respectively [101]. By comparison, only ~20% of the total PRO in commercial bovine milk comes from whey [23-25]. Camel's milk, a source growing in popularity but lacking in human physiological response data, is only ~15% whey [102]. Comprising whey are the PRO components β-lactoglobulin (~50-55%), α-lactalbumin (~20-25%), glycomacropeptide (~10-15%), immunoglobulins (~10-15%), serum albumin (~5-10%), lactoferrin (~1%), lactoperoxidase (<1%), and other minor proteins such as β-microglobulin, lysozyme, insulin-like growth factors and γ-globulins [23-25,103,104]. Of the aforementioned whey fractions, β-lactoglobulin may be especially important for stimulating MPS according to at least one recent study in older rats that compared the effects of leucine-rich PROs on postprandial MPS [105]. Notably, β-lactoglobulin, representing about 50-55% of the PRO in whey, is a major source of BCAA and is composed largely of the BCAA leucine - approximately 14.5% of β-lactoglobulin is made up of leucine residues. The potential, critical role of a PRO's leucine concentration on eliciting MPS has thus far only been observed in rats, however [106]. Interestingly enough, recent evidence also seems to indicate that native, or peptide-bound leucine, as opposed to free-form leucine may be utilized most efficiently [107]. This may imply that intact whey or its hydrolysated peptides may provide a superior MPS response than equivocal amounts of free leucine when consumed post-exercise. Similarly, whey is considered a high-quality PRO because of its abundance of EAA [23] which are necessary for stimulating protein synthesis and supporting muscle growth (Table 1) [108,109]. Whey is also classified as a "fast" PRO due to the rate at which its consumption leads to an increase in amino acid availability [10,79,110], which may be important for athletes and specifically valuable around workouts. The rate and concentration of amino acid availability may also be predictive of the MPS response [95], which may explain the superiority of whey versus soy or casein at stimulating MPS [11]. In the future, more research should be done comparing protein fractions in whey on MPS [105] and body composition [111], especially when combined with exercise.

**Table 1:** Approximate Essential Amino Acid Profile of Various Protein Sources

ESSENTIAL AMINO ACID	MILK PROTEIN ISOLATE	WHEY PROTEIN ISOLATE	WHEY PROTEIN HYDROL.	CASEIN	SOY PROTEIN ISOLATE	EGG PROTEIN
Isoleucine	4.4	6.1	5.5	4.7	4.9	5.7
Leucine	10.3	12.2	14.2	8.9	8.2	8.4
Lysine	8.1	10.2	10.2	7.6	6.3	6.8
Methionine	3.3	3.3	2.4	3.0	1.3	3.4
Phenylalanine	5.0	3.0	3.8	5.1	5.2	5.8
Threonine	4.5	6.8	5.5	4.4	3.8	4.6
Tryptophan	1.4	1.8	2.3	1.2	1.3	1.2
Valine	5.7	5.9	5.9	5.9	5.0	6.4

**Total BCAAs**	**20.4**	**24.2**	**25.6**	**19.5**	**18.1**	**20.4**
**Total EAAs**	**42.7**	**49.2**	**49.8**	**40.7**	**36.0**	**42.3**

Approximate concentration of essential and branched chain amino acids (EAA and BCAA, respectively) present within various forms of commercially available protein (g/100 g). Adapted from [27]. Casein is the average of reported values for Calcium Caseinate, Sodium Caseinate, and Potassium Caseinate; Whey Protein Isolate is the average of reported values for Ion-Exchange and Cross-Flow Microfiltrated Whey Protein Isolates. Hydrol. is hydrolysate.

## Whey protein supplements

Whey PRO is categorized, commercially, as either whey PRO concentrate (WPC), whey PRO isolate (WPI), or whey PRO hydrolysate (WPH, or hydrolyzed whey). WPC, or native whey, may contain 29% to 89% total protein by volume (g/100 g), with the remaining nutrient composition coming from carbohydrate (predominantly lactose) and lipid. However, the relatively small differences in lipid and carbohydrate concentrations between WPC's of high protein concentration (e.g., ≥70%), and WPI or a typical WPH, may not significantly affect the rate of gastric emptying and amino acid absorption as compared to what has been observed in response to WPI and WPH [112,113]. In fact, addition of milk fat may offer a slightly positive effect on post-exercise protein balance according to at least one study that compared the effects of whole versus fat-free milk [114]. However, to date, no data exists that has compared the acute absorption kinetics and MPS response, or long-term physiological adaptation from WPCs of varying concentrations to other WPCs, WPIs or WPHs. Interestingly enough, WPC-70 and WPC-80 (70% and 80% concentrations of protein, respectively) are the most common forms of whey PRO used within sports supplements, largely due to current raw material pricing and possibly improved taste characteristics when compared to either WPI or various forms of WPH [115]. Paradoxically, the vast majority of whey PRO research has utilized either WPI or WPH. WPI contains ≥90% total protein by volume and very little, or insignificant amounts of lactose and lipids. The low lactose content of WPI may be of importance for individuals with lactose intolerance, especially when large amounts of whey or other dairy products are consumed daily. Also, WPI may be more suitable than WPC when used in combination with a very-low-carbohydrate (ketogenic) diet.

PRO can also be enzymatically or otherwise hydrolyzed (i.e., partially "pre-digested") during manufacturing, producing smaller peptides which may accelerate the absorption and utilization of amino acids [116]. Accordingly, as discovered by Koopman et al. [95] when comparing intact casein versus casein hydrolysate, the faster rate of amino acid appearance and greater peak amino acid concentration obtained from casein hydrolysate *may *(*p*-value was reported as *p *= 0.10) combine to accentuate the MPS response to exercise [95]. However, the data comparing whey PRO to its hydrolysate is even less clear. In fact, whey may already be an effectively fast-acting source such that hydrolysis does not further augment its gastric emptying [113] or substantially accelerate the increase in blood amino acid concentrations [52]. One limitation to our current understanding of the differences between native whey and its hydrolysate is that all hydrolysis does not yield the same homogenous PRO containing end-product. Instead, degree of hydrolysis varies considerably and is rarely reported in studies and almost never on product labels; nor is the concentration of PRO fragments by molecular weight generally disclosed within the literature. For example, to possibly optimize PEPT-1 di- and tri-peptide transporters for rapid amino acid absorption and to eliminate allergenicity for some of the large dairy proteins, it has previously been recommended that the majority of PRO fractions be ≤ 0.5 kDa in molecular weight [117]. It could be argued, however, that such extensive hydrolysis may ameliorate possible positive effects from some of the larger whey peptides [23]. Despite absorption kinetics of whey versus its hydrolysates being equivocal at the moment, WPH has been shown to yield a significantly greater insulin response compared to either WPI or WPC [52,113]; and, as mentioned earlier, at least one acute training study in humans has reported improved recovery from WPH versus its WPI source material [46]. Thus, it is possible that WPH may elicit an enhanced effect on muscle hypertrophy when combined with chronic resistance training. Indeed, data collection has recently concluded within our laboratory at the University of Oklahoma to address this very question. Also, see Manninen [116] for a recent review on PRO hydrolysates.

## Are the effects of whey protein simply due to its constituent essential amino acids?

As alluded to previously, the effects of whey on muscle adaptations may not be solely dependent upon its EAA concentration. A study that may confuse that argument was one in which the consumption of 15 g of EAA almost doubled the muscle protein balance in elderly subjects, compared to consuming whey [118]. Of note, however, the subjects consumed isocaloric amounts of either EAA or whey, and thus the whey trial consisted of ~50% less EAA. From this study, it would appear that whey may be *energetically *less efficient than consumption of its constituent EAA for increasing muscle protein balance, at least for elderly individuals, as its constituent non-essential amino acids do not seem to be as important in enhancing protein balance/synthesis [3,108,109]. Contrastingly, acute whey PRO ingestion (15 g), under resting conditions and in elderly men and women, resulted in greater muscle protein balance than consumption of its constituent EAAs (6.72 g) or non-essential amino acids (7.57 g) [10]. This result may suggest that something other than EAAs within whey are important for muscle hypertrophy. For example, it is possible that via the PEPT-1 cotransporters' high capacity, low specificity rate of transport, and an apparent increased transport affinity for L-valine bound peptides, that the bound form of an EAA may be more efficiently utilized than when delivered in its free-form [119]. Similarly, new discoveries continue to surface regarding bioactive peptides present within dairy, and specifically in whey that may facilitate improved recovery and antioxidative capacity to support physiological adaptations to exercise [104]. However, possible long term superiority of whey compared to its constituent amino acids (all, or just its EAAs) is not known.

## Whey protein and leucine

It is probable that the most important component in whey, for increasing protein synthesis and skeletal muscle size, is its high concentration of the BCAA, leucine (see Table 1). Leucine, acting as a signaling molecule in the mTOR cascade [61,120], has been shown to be a critical amino acid for increasing skeletal muscle protein synthesis, both *in vitro *[58] and *in vivo *in humans [39] and rats [59,105]. Leucine may also be involved in suppressing muscle protein degradation, according to investigations *in vitro *[58] and *in vivo *in humans [121]. However, if recent data involving rodents can be duplicated in humans, leucine concentrations from whey may only affect peak activation of skeletal MPS but not the duration of MPS or duration of mTOR signaling [106]. Similarly, despite the positive effects of leucine *per se*, it likely is not the sole factor responsible for whey-induced adaptations to resistance training. For example, adding leucine to intact protein has been shown to offer little, if any, effect on protein synthesis and protein balance when consumed after resistance exercise [7,122,123], and 7.5 g/d of supplemental leucine for three months was not shown to increase muscle mass or strength in elderly males [124]. However, the latter study did not include training, or may be indicative of what Moore et al. [9] found regarding compartmental/fraction-specific protein synthesis in response to PRO feeding alone versus in combination with resistance exercise. Also, results by Katsanos et al. [39] imply that a higher leucine threshold is necessary to stimulate protein synthesis in older adults. And lastly, it is possible that free-form versus native/bound leucine may yield varying efficacy. Thus, there still exists a need to adequately assess the long-term effects of supplemental whey versus leucine, in combination with resistance exercise, on MPS and muscle hypertrophy.

## Conclusion

Most, but not all studies have shown that supplementation of whey alone or with carbohydrates immediately after and possibly before and during resistance exercise can enhance the muscle hypertrophy response to resistance training in healthy adults. Such a response seems to at least be the case when comparing the effects of whey versus a non-energetic, or carbohydrate or soy protein alternative. Some studies also suggest that whey may enhance recovery from heavy exercise and possibly decrease muscle damage and soreness. This could, over time, enhance training adaptations by way of increasing training volume or reducing the potential for over-reaching/over-training. However, much is yet to be understood about the effects of whey protein on the physiological response to resistance training. Future research should look to assess the effects of dose response and timing of protein ingestion, compare the effects of various forms/fractions of whey as well as compare the effects of whey to other protein sources, and assess the effects of whey protein in muscle atrophying diseases, to name a few.

## Abbreviations

BCAA: branched-chain amino acids; CHO: carbohydrates; EAA: essential amino acids; MPB: muscle protein breakdown; MPS: muscle protein synthesis; mTOR: mammalian target of rapamycin; NPB: net protein balance; PRO: protein; WPC: whey protein concentrate; WPH: whey protein hydrolysate; WPI: whey protein isolate

## Competing interests

The authors declare that they have no competing interests.

## Authors' contributions

JJH: Involved in analyses and interpretation of reference material, drafting and revising the manuscript, and giving final approval to the version to be published.

CML: Involved in analyses and interpretation of reference material, drafting and revising the manuscript, and giving final approval to the version to be published.

JRS: Involved in analyses and interpretation of reference material, drafting and revising the manuscript, and giving final approval to the version to be published.
